# Variability in Hemoglobin Levels and the Factors Associated with Mortality in Hemodialysis Patients: A 78-Month Follow-Up Study

**DOI:** 10.3390/ijerph18031078

**Published:** 2021-01-26

**Authors:** Natalino Salgado Filho, Joyce Santos Lages, Dyego José de Araújo Brito, Elton John Freitas Santos, Alcione Miranda dos Santos, Francival Leite de Souza, Vinícius Giuliano Gonçalves Mendes, Giselle Andrade dos Santos Silva, Erika Cristina Ribeiro de Lima Carneiro, Monique Pereira Rêgo Muniz, Gyl Eanes Barros Silva, Ricardo de Castro Cintra Sesso

**Affiliations:** 1 Renal Division, Federal University of Maranhão, São Luís 65020-070, Brazil; joyce_lages@uol.com.br (J.S.L.); djabrito@uol.com.br (D.J.d.A.B.); eltonfreitas86@yahoo.com.br (E.J.F.S.); alcione.miranda@gmail.com (A.M.d.S.); francival.souza@uol.com.br (F.L.d.S.); vggmendes@gmail.com (V.G.G.M.); giselle.silva@huufma.br (G.A.d.S.S.); erikacarneiro0204@gmail.com (E.C.R.d.L.C.); moniqueprmuniz@gmail.com (M.P.R.M.); gyleanes@fmrp.usp.br (G.E.B.S.); 2 Pathology Division, University of São Paulo (USP), Ribeirão Preto 14049-900, Brazil; 3 Discipline of Nephrology, Paulista School of Medicina, Federal University of São Paulo, São Paulo 04023-062, Brazil; rsesso@unifesp.br

**Keywords:** hemoglobin, erythropoiesis-stimulating agents, mortality, hemodialysis

## Abstract

Some studies have described that when the hemoglobin levels of chronic kidney disease (CKD) patients change, especially in those taking erythropoiesis-stimulating agents (ESA), they are associated with unfavorable outcomes such as increased morbidity and mortality, mainly due to cardiovascular events. This prospective cohort study included patients with end-stage renal disease currently undergoing hemodialysis. The initial 6-month clinical evaluation provided data of the variability in hemoglobin, associated blood parameters, and the use of erythropoietin. Subsequently, the patients were followed up for 78 months to evaluate mortality-associated factors. In total, 133 patients completed the 6-month follow-up with a mean age of 47.1 (±13.2) years. The majority were women (51.9%). Six-month hemoglobin levels were as follows: always low (18.0%), intermediate/target (1.5%), always high (0.8%), low-amplitude fluctuation/Hb low (*n* = 37; 27.8%), low-amplitude fluctuation/Hb high (13.53%), and high-amplitude fluctuation (38.6%), among end-stage renal disease patients. At the end of 78 months, 50 (37.6%) patients died; 70% of deaths were attributed to cardiovascular etiologies. A high variability was observed in hemoglobin levels, which was not associated with mortality. Among all the variables evaluated, age, erythropoietin dose, and transferrin saturation were associated with a higher mortality. Thus, this study suggests that greater attention to erythropoietin doses and transferrin saturation levels may improve the survival of dialysis patients.

## 1. Introduction

The management of anemia has become central to the treatment of patients with chronic kidney disease (CKD) under dialysis. Observational studies show that patients experience considerable changes in hemoglobin levels over time, with only 5% of them being able to maintain normal levels (Hb 11–12 g/dL) during a 6-month period [1,2,3]. Ebben et al. reported that only 10.3% of patients with stage 5 chronic kidney disease had stable hemoglobin levels during a 6-month study duration and only 6.5% of patients had their hemoglobin levels in the target range of 11–12 g/dL [4].

When the hemoglobin levels of CKD patients change, especially in those taking erythropoiesis-stimulating agents (ESA), they are associated with unfavorable outcomes such as increased morbidity and mortality, mainly due to cardiovascular events [5,6], as described in a meta-analysis published by Phrommintikul et al. who observed a significant increase in the risk of mortality when the hemoglobin level was between 12 and 16 g/dL [7].

In a prospective cohort study with 432 patients with CKD on dialysis, Foley et al. demonstrated that lowering the hemoglobin level by 1 g/dL was independently associated with the dilation of the left ventricle, and with the development or recurrence of heart failure [8]. Left ventricular hypertrophy is a survival factor for CKD and is present in the majority of patients who start on dialysis [9]. Low serum hemoglobin level is an independent predictor of left ventricular hypertrophy even in patients with mild to moderate renal dysfunction [10].

A high risk of death and/or adverse events in patients with hemoglobin levels outside the target range and in those with high-amplitude hemoglobin fluctuations was confirmed by the research of Kuragano et al. [11], who also found an association between consistently high serum ferritin levels and high-amplitude ferritin fluctuations and bad prognosis. More recently, a prospective study of 169 patients over a period of 12 months demonstrated that high hemoglobin variability is an independent risk factor for cardiovascular mortality in hemodialysis patients and might influence cardiac function [12]. Major predictors of hemoglobin variability seem to be inflammation and duration of anemia [13].

Hence, in accordance with the above associations between changes in hemoglobin levels and the risks for cardiovascular events, we aimed to evaluate the variability of hemoglobin and the factors associated with the mortality of patients on hemodialysis during a 78-month follow-up period.

## 2. Materials and Methods

### 2.1. Study Protocol

This is a prospective cohort study, which included patients with end-stage renal disease (ESRD) undergoing hemodialysis at two centers in São Luís, Maranhão, Brazil, from January 2009 to January 2016. The study was divided into two stages: the 1st stage (inclusion and follow-up for six months to assess hemoglobin variability and associated factors), and the 2nd stage (follow-up for seventy-eight months to assess patient survival and identifying mortality-related factors). In the first stage, the patients answered a standardized questionnaire with sociodemographic data and clinical history, after which they underwent monthly laboratory assessments (January to June 2009). In the 2nd stage (July 2009 to January 2016), the initial group was monitored for outcome assessment (survival × mortality), with visits to the centers every 6 months.

### 2.2. Patients

Patients were selected from two hemodialysis centers (an outpatient and a hospital center) at São Luís, Maranhão, Brazil, which treated 390 patients. Patients were selected from a nominal list supplied by each center, after applying the inclusion and exclusion criteria, which are presented in Table 1.

Patients who met the inclusion criteria (*n* = 165) were informed about the study and those who agreed to participate signed an informed consent form. In the first 6-month follow-up, patient exclusions happened due to death, withdrawals from the study, entry into clinical studies, transfer from the treatment center, or transplantation. One hundred and thirty-three patients completed the 1st study stage and entered into the follow-up stage for 78 months.

### 2.3. Laboratory Assessments

In the second session of hemodialysis for the first week of each month, biological samples were collected to prevent the interference of hemodilution in the interpretation of laboratory tests. During these 6 months, high-sensitivity C-reactive protein (hsCRP), complete blood count, iron, ferritin, transferrin, and reticulocytes were measured every month. The samples were processed in the clinical research laboratory of our institution.

### 2.4. Definitions

The target level of hemoglobin was taken to be 10–12 g/dL. For identifying the factors influencing the change in hemoglobin levels, we used the Kidney Disease Improving Global Outcomes (KDIGO) definitions [11]. The monthly Hb values for each patient were classified as: low (<10 g/dL), intermediate (10 to 12 g/dL), or high (>12 g/dL). To evaluate the variation, patients were separated into six groups: always low; intermediate/target; always high; low-amplitude fluctuation/low Hb (BA/HbB); low-amplitude fluctuation/high Hb (BA/HbA); and high-amplitude fluctuation (AA), as described by Ebben et al. [4] (Appendix A). Transferrin saturation levels of 30–55% were considered normal. Systolic Blood Pressure (SBP) and Diastolic Blood Pressure (DBP) were evaluated by average measures of each first register of blood pressure of the first hemodialysis session of the month during the follow up.

### 2.5. Statistical Analysis

In the descriptive analysis, the numerical variables were presented as mean and standard deviation (mean ± SD) or median (minimum and maximum values), and the categorical variables as frequency and percentage. The Shapiro–Wilk test was used to verify the normality of the numerical variables, and the Chi-square test was used for comparison of the proportions. The Cox regression model was used to identify mortality-associated factors. The multivariate model included the variables with *p* < 0.10 in the univariate analysis. Only the variables with *p* ≤ 0.05 remained in the final model. Data were processed using Stata 12.0 (StataCorp, College Station, TX, USA).

### 2.6. Ethical Considerations

This study was approved by the Research Ethics Committee of the University Hospital of the Federal University of Maranhão (CEP/HUUFMA) under protocol number 004150/08-50, in accordance with the norms for research on human beings.

## 3. Results

One hundred and thirty-three patients completed the initial study period and entered into the monitoring stage. The mean age of this group was 47.1 (±13.2) years, with women occupying the majority (51.9%). Hypertension was the most common etiology of ESRD patients followed by chronic glomerulonephritis. The average time of hemodialysis was 41.8 (±41.3) months. Approximately 27% of the patients had a cardiovascular diagnosis. The baseline characteristics of the sample are shown in Table 2.

There was no difference between doses of erythropoietin used between patients aged 60 years and over (22 patients, 16.5%) and patients under 60 years (111 patients, 83.5%).

During the initial 6-month follow-up, the levels of hemoglobin observed in the study cohort were: always low (*n* = 24; 18.0%); intermediate/target (*n* = 2; 1.5%); always high (*n* = 1; 0.8%); low-amplitude fluctuation/Hb low (*n* = 37; 27.8%); low-amplitude fluctuation/Hb high (*n* = 18; 13.53%); and high amplitude fluctuation (*n* = 51; 38.6%).

After 78 months of follow-up, 18 transplants were recorded and 50 (37.6%) patients died, with cardiovascular causes being responsible for 70% of deaths. Sudden death was the main cause (34.3%) of cardiovascular death (Table 3).

**Figure 1:**
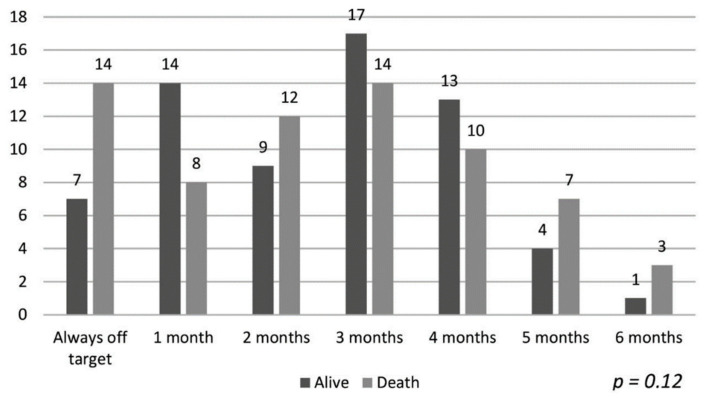
Target hemoglobin time and mortality.

**Figure 2:**
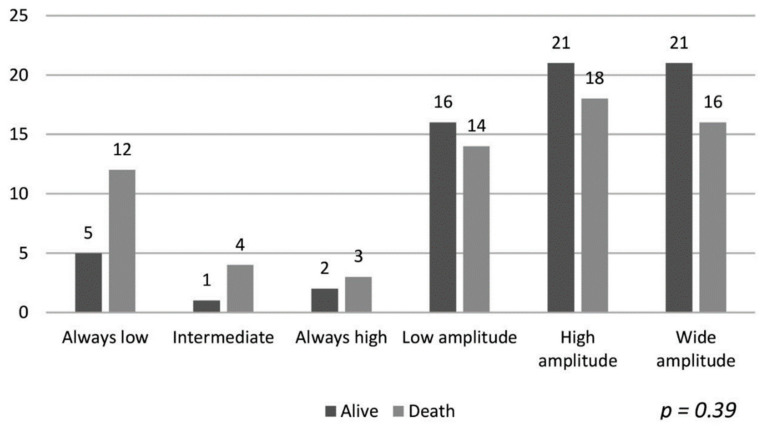
Patterns of variability in hemoglobin levels (according to Ebben et al. [4]) and mortality.

The data of the patients maintaining hemoglobin in the target range and their association with mortality are shown in Figure 1. Despite a longer duration of maintaining target hemoglobin, the association with mortality was not statistically significant (*p* = 0.12). Similarly, the hemoglobin variability patterns described by Ebben et al. were not associated with overall group mortality [4], as shown in Figure 2 (*p* = 0.39).

Overall mortality was associated with age (HR 1.02, *p* = 0.01), diabetes mellitus (HR 2.01, *p* < 0.01), systolic blood pressure (SBP) >140 mmHg (HR 1.78, *p* = 0.04), hemoglobin levels above median (HR 0.57, *p* = 0.03), and erythropoietin dose above the median (HR 1.94, *p* = 0.01) in the univariate analysis (Table 4). There was no association between ferritin level and death in this study. The multivariate Cox regression model analysis showed that only age and erythropoietin dose above the median were significantly associated with overall mortality (Table 5).

Regarding cardiovascular mortality, there was a significant association with diabetes mellitus (HR 2.69, *p* = 0.01), SBP >140 mmHg (HR 2.88, *p* = 0.01), hemoglobin levels above the median (HR 0.41, *p* = 0.02), and transferrin saturation index <30% or >55% (HR 2.55, *p* = 0.01) in the univariate analysis (Table 6). In the multivariate model, only transferrin saturation index <30% or >55% maintained an association with statistical significance (HR 2.39, *p* = 0.04).

## 4. Discussion

This study aimed to evaluate the variability of hemoglobin and the factors associated with the mortality of patients on hemodialysis and found that among all the variables evaluated, age, erythropoietin dose, and transferrin saturation were associated with a higher mortality in patients with stage 5 chronic kidney disease. Patients with ESRD have an increased risk of mortality, about 10–20 times higher than other patient populations [14], and cardiovascular complications are the main cause of death of patients under dialysis [15]. Factors such as anemia, mineral bone disorder, inflammation, systemic arterial hypertension, hypervolemia, and malnutrition, alone or together, are responsible for the high cardiovascular mortality [16]. The last census of the Brazilian Society of Nephrology (Sociedade Brasileira de Nefrologia) reported a progressive increase in the mortality of patients under dialysis, reaching 18.2% in 2016 [17], close to the values observed in the USA [18] and Europe [19], where these rates were always higher than those in Brazil. In this study, during a 78-month follow-up period, 50 patients died (mortality rate of 6.9% patients/year), lower than that found in the annual survey of the BSN, probably related to the quality of care provided to patients. 

The relationship between hemoglobin levels, use of ESA, and mortality has been studied previously, especially among patients with chronic kidney disease [20,21,22]. The TREAT [23] study showed no statistical association between cardiovascular and renal outcomes with the correction of anemia using darbepoetin or placebo. The CREATE [24] and CHOIR [25] studies also showed no reduction in the mortality of ESRD patients who received an erythropoietin dose to maintain hemoglobin levels above 11–12 g/dL, which are currently recommended by the KDIGO [14]. Moreover, the CHOIR [25] study was finalized before the period laid down by the higher tendency for mortality in the group with higher hemoglobin. In contrast, a meta-analysis of nine studies with 5143 patients, performed by Phrommintikul et al. [7], indicated an increased risk of all-cause mortality among patients with high hemoglobin levels treated with EPO; the same study showed similar incidences of acute myocardial infarction in the patients. Ogawa et al. studied the relationship between responsiveness to ESA and outcome in 320 patients undergoing hemodialysis [26] and found a higher risk of mortality among patients with hemoglobin levels <10 g/dL and a dose of ESA >120 IU/kg/week. Likewise, our study showed that the use of erythropoietin at doses above the median of the group could be associated with overall mortality.

Epidemiological studies revealed an increase in the elderly population on dialysis [27]. Regardless of the presence of renal dysfunction, elderly individuals have a high risk of cardiovascular complications and mortality [28]. A recent meta-analysis of 23 studies performed by Ma et al. including 86,915 individuals undergoing hemodialysis [29] reported that age increased the all-cause mortality risk. Similarly, Myers et al. concluded that age was also associated with the higher mortality of patients on dialysis [30], but there is a strong influence from blood pressure levels, as lower systolic and diastolic blood pressure were associated with higher mortality in patients aged 50 years and above. In our study, age was associated with increased all-cause mortality, regardless of other factors. It is worth mentioning that the mean age of the Brazilian patients who initiate hemodialysis was considerably lower (41 years) when compared to, for example, European patients, who initiate hemodialysis mostly at an age of over 65 years [31]. Thereby, the younger age of our patients has probably played an important role in the good survival observed.

Although the life expectancy of women is higher than that of the general population [32], several studies show similar survival rates for both genders with respect to ESRD patients on hemodialysis [33]. The difference in life expectancy for women with ESRD on hemodialysis as compared to those without the disease is attributed to the low doses of erythropoietin administered during dialysis, which increases their chances of developing anemia along with comorbidities such as low bone mineral densities that necessitate treatment with temporary vascular access [33]. A retrospective cohort study conducted with 28,971 Canadian patients showed that women aged <45 years presented a 31% higher risk of death when compared to men of the same age [34]. The relationship between female sex and risk of mortality in patients on dialysis was also recently published by Ma et al. [29]. Comparing the sexes, the authors reported a 41% higher risk of death among women due to cardiovascular causes. These results differ from those presented in this study, which found lower mortality from all causes among women, possibly influenced by the low mean age of the group, associated with the appropriate doses of dialysis and the presence of definitive vascular access in all patients.

The association between transferrin saturation and mortality was reported previously. In a cohort study using data from the NHANES study, Mainous et al. reported that levels of transferrin saturation greater than 55% were associated with increased mortality from all causes [35]. Another study, published by Well et al. observed a combined effect of the same transferrin saturation values described above and high levels of LDL (>160 mg/dL) on mortality in a healthy North American population [36]; the authors ascertained that the increased oxidation of LDL by iron makes lipoprotein more atherogenic, which increases cardiovascular mortality. In our study, which included only CKD patients on hemodialysis, elevated (<55%) or low (<30%) transferrin saturation was associated with higher cardiovascular mortality. 

The main limitation of the present study is the small number of patients from one region; however, statistical tests were used to calculate a significant sample size of the population studied.

Another limitation of this study is that it is not possible to affirm if the presence of worse clinical conditions in older patients is the real factor determining bad prognosis in this population. Weinhandl et al. [37] examined three groups of Medicare data: prevalent dialysis patients on 1 July 2006 (*n* = 133,246); prevalent dialysis patients on 1 July 1996 (*n* = 78,602); and incident patients between 1 January 2005 and 30 June 2006 (*n* = 24,999). Because disease severity factors both influence Hb level variability and predict death, the objective of the study was to determine whether adjustment for disease severity would decrease or eliminate the association between Hb level variability and mortality risk described in previous studies, such as those of Yang et al. [38]. The conclusion was that after adjustment for confounding disease severity, evidence supporting an association between interpatient Hb level variability and mortality is weak and inconsistent. In this study, there was no difference between the doses of erythropoietin used between patients aged 60 years and over and patients aged under 60 years. However, we cannot say that the mortality difference between the youngest and the oldest was due only to the variability of hemoglobin.

Moreover, since inflammation seems to be one of the major predictors of hemoglobin variability [11,13] and we did not analyze the influence of hemoglobin variability, iron biomarkers values, and inflammatory markers during the observational time of the study, it is not certain that patients have had no change in hemoglobin variability categories during their 6 years of follow-up. Conversely, they could have become either iron-depleted or iron-overloaded during this period or inflamed, which could confound the results. 

The strength of the study was the long period of follow-up that allowed an evaluation of the outcomes of the long-term factors related to anemia. We thereby conclude that transferrin saturation (<30% or >55%) and high doses of erythropoietin are related to increased mortality in patients undergoing hemodialysis.

## 5. Conclusions

No association was observed between hemoglobin levels or its variations and the mortality of patients during a 78-month follow-up period. Age and erythropoietin dose above the median were associated with increased all-cause mortality and the levels of transferrin saturation displayed an association with cardiovascular mortality. Women were associated with a lower occurrence of deaths. We also ascertain that the overlap of risk factors may affect mortality among end-stage kidney disease patients on dialysis and require strict monitoring and treatment to reduce the occurrence of adverse outcomes, such as cardiovascular events and death. More studies should be developed in order to understand the mechanisms involved in these results, so that they can be modified in clinical practice. 

## Figures and Tables

**Table 1:** Inclusion and exclusion criteria.

Inclusion Criteria	Exclusion Criteria
ESRD patients in a regular hemodialysis program for at least 3 months; Regular use of erythropoiesis stimulating agents; Age greater than or equal to 18 years; Patients who signed the informed consent form.	Patients with active malignant disease, chronic liver disease, severe cognitive deficit, hematological diseases, and secondary non-controlled hyperparathyroidism (PTH ≥ 600 pg/dL); Patients who received blood transfusion in the 3 months prior to the beginning of the study; The presence of significant acute or chronic bleeding; Deficiency of folic acid and vitamin B12; Major elective surgery in the 3 months prior to the study; Temporary access to dialysis; Participation in clinical trials.

Legend: ESRD, End stage renal disease; PTH, parathyroid hormone.

**Table 2:** Characteristics of the studied sample (*n* = 133).

Parameter	Values
Age (years ± SD)	47.1 ± 13.2 years
Sex	
Male	64 (48.1%)
Female	69 (51.9%)
Ethnicity	
White	15 (11.3%)
Black	30 (22.6%)
Mixed-race	72 (54.2%)
Other	16 (12.1%)
Duration of hemodialysis (months ± SD) ^a^	41.8 ± 41.3 months
CKD etiology	
Systemic arterial hypertension	48 (36.1%)
Diabetes Mellitus	23 (17.3%)
Chronic Glomerulonephritis	35 (26.3%)
Other causes	27 (20.3%)
Co-morbidities	
Systemic arterial hypertension	119 (89.4%)
Diabetes mellitus	43 (32.3%)
Cardiovascular Disease	37 (27.8%)
Smoking	8 (6.0%)
Alcohol consumption	11 (8.3%)
BMI (kg/m^2^)	
<18.5	19 (14.3%)
18.5–24.9	86 (64.7%)
25–29.9	25 (18.8%)
≥30	3 (2.2%)
SBP (mmHg)	138.7 ± 22.9
DBP (mmHg)	82.5 ± 10.0
Hemoglobin (g/dL)	10.9 ± 1.9
Reticulocytes (%) ^b^	34.4 (4.5–111.2)
Ferritin (ng/mL) ^b^	470.5 (18.2–2590.7)
Serum iron (µg/dL) ^b^	65 (16–202)
Transferrin saturation (%) ^b^	38.3 (10.2–128.7)
hsCRP (mg/L)	
<1	112 (84.2%)
1–3	16 (12.0%)
≥3	5 (3.8%)
Weekly Dose of EPO (IU) ^b^	9333.3 (3333.3–24,000)

Legend: CKD—chronic kidney disease; BMI—body mass index (in kg/m^2^); SBP—systolic blood pressure; DBP—diastolic blood pressure; hsCRP—high-sensitivity C-reactive protein (in mg/L); EPO—erythropoietin (in IU). SD—standard deviation. ^a^ mean, ^b^ median.

**Table 3:** Outcomes observed over 78 months in the sample studied (*n* = 133).

Parameter	*n* (%)
**Outcome**	
Death	50 (37.6)
Transplant	18 (13.5)
Survival after hemodialysis	65 (48.9)
**Type of Death**	
Cardiovascular	35 (70.0)
Infection	7 (14.0)
Other	8 (16.0)
**Cardiovascular mortality**	
Sudden death	12 (34.3)
Stroke	9 (25.7)
Acute myocardial infarction	7 (20.0)
Arrhythmia	6 (17.1)
Congestive heart failure	1 (2.9)

**Table 4:** Factors associated with mortality in the long term (78 months): univariate Cox regression model.

Variables	Hazard Ratio	*p*-Value
Female sex	0.62	0.06
Age	1.02	0.01
Diabetes mellitus	2.01	<0.01
SBP > 140 mmHg	1.78	0.04
Mean hemoglobin	0.84	0.05
Transferrin saturation <30% and >55%	1.66	0.05
hsCRP > 3 mg/L	2.06	0.05
EPO dose > median	1.94	0.01

Legend: SBP—systolic blood pressure (mmHg); hsCRP—high-sensitivity C-reactive protein (in mg/L), EPO—erythropoietin.

**Table 5:** Factors associated with the overall mortality over the long term (78 months): multivariate Cox regression analysis.

Variables	Hazard Ratio	*p*-Value
Female sex	0.53	0.01
Age	1.02	0.02
EPO dose > median	1.94	0.01

Legend: EPO—erythropoietin.

**Table 6:** Risk factors associated with cardiovascular mortality in the long term (78 months): univariate Cox regression analysis.

Variables	Hazard Ratio	*p*-Value
Female sex	0.59	0.15
Age	1.01	0.55
Diabetes mellitus	2.69	0.01
SBP > 140 mmHg	2.88	0.01
Hemoglobin > median	0.41	0.02
Transferrin saturation <30% and >55%	2.55	0.01
HsCRP > 3 mg/L	1.80	0.24
EPO dose > median	1.69	0.16

## Data Availability

The data presented in this study are available on request from the corresponding author. The data are not publicly available due to their containing information that could compromise the privacy of research participants.

## Appendix A

Ebben et al. (2006) [4] evaluated the frequency with which hemodialysis patients maintain stable hemoglobin levels below, within, and above the Centers for Medicare and Medicaid Services target range and assessed patterns of hemoglobin level change that resulted in large fluctuations across the target range during a 6-month period. They studied 152,846 hemodialysis patients and divided them into six patient groups, which were defined on the basis of patterns of hemoglobin level fluctuation (Table 1). Patients who were classified in the low-amplitude fluctuation with low hemoglobin levels and low-amplitude fluctuation with high hemoglobin levels groups showed fluctuations in their hemoglobin levels near the lower and upper levels of the target range, such that their hemoglobin levels crossed one of the boundaries at 11 or 12.5 g/dL during the 6-month period. Patients who were classified in the high-amplitude fluctuation group showed large fluctuations in their hemoglobin levels, such that they crossed both the upper and the lower boundaries of the target range.

Ebben et al. concluded that hemoglobin levels in almost 90% of patients are in some degree of flux at any point in time, and the fluctuation is highly associated with clinical complications, as shown below in Table A1.

**Table A1:** Patterns of hemoglobin level fluctuation and association with clinical complications and provider practices.

Patterns of Hemoglobin Level Fluctuation	Frequency (%)	Hospital Admission (%)	Admission for Infection (%)	Average Length of Hospital Stay (d)	Average Comorbidity (n)
Consistently low (<11 g/dL)	1.8	69.2	29.5	12.7	2.4
Consistently target range (11 to 12.5 g/dL)	6.5	25.3	6.2	1.9	1.1
Consistently high (≥12.5 g/dL)	2.0	29.8	7.4	2.2	1.2
Low-amplitude fluctuation with low hemoglobin levels	21.3	51.1	17.6	6.5	1.8
Low-amplitude fluctuation with high hemoglobin levels	28.9	33.5	9.3	2.8	1.3
High-amplitude fluctuation	39.5	54.0	17.7	6.4	1.8

Reproduced with permission from Ebben et al. [4], American Society of Nephrology—Clinical Journal (online); published by American Society of Nephrology, 2006.
